# Two cases of acute pancreatitis occurring during therapeutic hypothermia for severe brain injury: case report and literature review

**DOI:** 10.3389/fmed.2026.1737244

**Published:** 2026-03-16

**Authors:** Rui Lu, Junhua Yan, Shuai Wang, Mengsha Yu

**Affiliations:** Department of Critical Care Medicine, Affiliated Hangzhou First People’s Hospital, Westlake University School of Medicine, Hangzhou, China

**Keywords:** acute pancreatitis, neurointensive care, severe brain injury, therapeutic hypothermia, valproate sodium

## Abstract

**Background:**

Therapeutic hypothermia is increasingly utilized in the management of severe brain injury. While lowering the target temperature can improve neurological outcomes, it also raises the incidence of treatment-related complications. Acute pancreatitis occurring during therapeutic hypothermia is exceptionally rare. Our center encountered two such cases within a short period, prompting this report to enhance clinical awareness.

**Case presentation:**

Case 1: A 75-years-old female with intracerebral hemorrhage received therapeutic hypothermia (target 34 °C). On the second day, she developed acute pancreatitis, which resolved after hypothermia was discontinued. Case 2: A 62-years-old male with post-thrombectomy cerebral edema underwent hypothermia (33 °C–34 °C). Similarly, acute pancreatitis emerged on the second day and improved upon rewarming. In both cases, common etiologies of pancreatitis were excluded. The temporal association suggested a possible link to hypothermia.

**Conclusion:**

Acute pancreatitis during therapeutic hypothermia is exceedingly rare. Its cause is likely multifactorial, involving factors such as hypothermia itself and stress responses. Therefore, clinicians should be vigilant about this potential complication during therapeutic hypothermia to avoid delays in diagnosis and treatment.

## Introduction

Primary brain injury triggers a cascade of secondary damage–neuroinflammation, excitotoxicity, oxidative stress, and apoptosis–that exacerbates neurological deficit. Therapeutic hypothermia is thought to mitigate these processes by reducing cerebral metabolic rate, thereby decreasing lactate and free radical production, limiting oxidative stress, and preserving neuronal integrity (1). Since its introduction by Busto et al. in the 1980s (2), the neuroprotective potential of mild-to-moderate hypothermia (32 °C–35 °C) has gained considerable attention.

As the application of therapeutic hypothermia expands, so does the recognition of its complications. Common adverse effects include cardiac arrhythmias, pneumonia, reduced gastrointestinal motility, electrolyte disturbances, and deep vein thrombosis (3, 4). In contrast, acute pancreatitis during therapeutic hypothermia is exceedingly rare. Our center observed two consecutive cases of acute pancreatitis in patients undergoing therapeutic hypothermia for severe brain injury, both of which prolonged hospital stay and raised clinical concern. This report aims to present these cases, discuss potential mechanisms, and highlight the need for vigilant monitoring.

## Case 1

### Patient information

A 75-years-old female with a history of hypertension and diabetes was admitted at 22:00 on October 10, 2024, presenting with “sudden loss of consciousness for 4 h.” While walking, the patient experienced a sudden onset of dizziness, followed by severe vomiting. She then became unconscious and unresponsive, accompanied by profuse sweating, pallor, and fecal and urinary incontinence. An ambulance was called, and a head CT scan at a local hospital indicated “cerebellar hemorrhage.” The patient was subsequently transferred to our Advanced Stroke Center.

### Clinical findings

**FIGURE 1:**
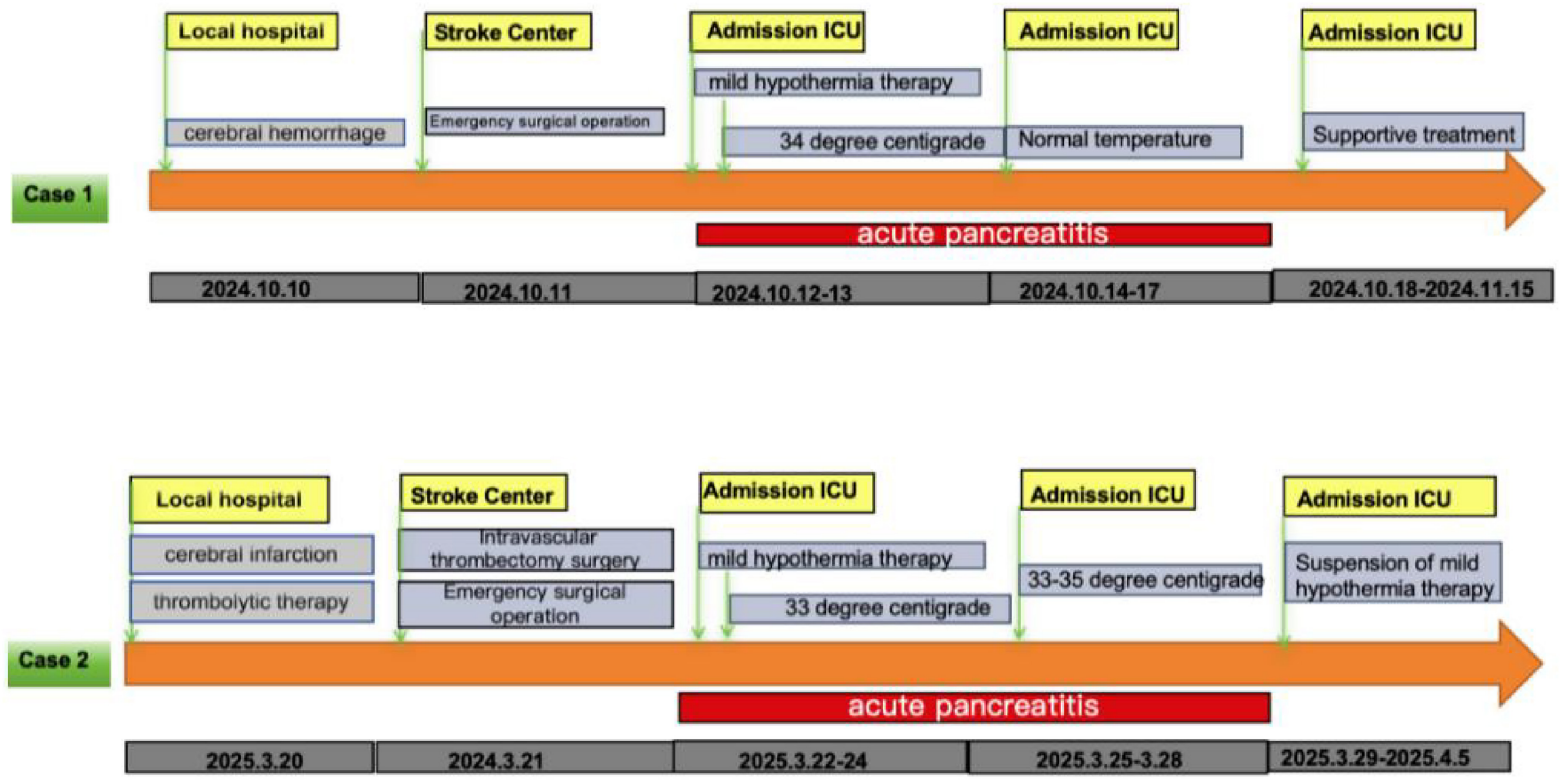
The treatment process of two cases.

Upon arrival at our Advanced Stroke Center, cranial CTA revealed: moderate stenosis in the M1 and M2 segments of the left middle cerebral artery, cerebellar hemorrhage, subarachnoid hemorrhage, and hematoma in the third ventricle, fourth ventricle, and bilateral lateral ventricles. After consultation with a neurosurgeon, the patient underwent emergency surgery: “Evacuation of Cerebellar Hematoma + Posterior Fossa Decompressive Craniectomy + External Ventricular Drainage.” Postoperatively, she was transferred to the ICU for advanced life support (Figure 1).

### Diagnostic assessment

Upon ICU admission, she was diagnosed with intracerebral hemorrhage and cerebral edema.

### Therapeutic intervention

**FIGURE 2:**
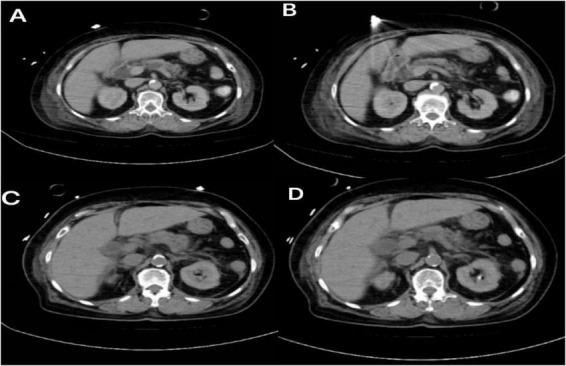
Representative non-contrast abdominal computed tomography images **(A–D)** of Case 1 demonstrate diffuse pancreatic swelling, along with edematous change in the duodenal wall near the bulb.

The patient was comatose with a Glasgow Coma Scale (GCS) score of 3. Following a comprehensive assessment of organ function, therapeutic hypothermia was initiated. Based on cerebral blood flow monitoring, the target body temperature was set at 34 °C. On October 12, a nasojejunal feeding tube was placed to initiate enteral nutrition. On October 13, physical examination revealed abdominal distension, and bladder pressure monitoring measured 21 cmH2O, prompting suspension of enteral nutrition. After administering an enema, laboratory tests showed a serum amylase level of 811 U/L (reference range: 35–135 U/L) and a lipase level of 476 U/L (reference range: 13–60 U/L). Triglyceride levels were 1.05 mmol/L (reference range: <1.70 mmol/L). Bedside ultrasound showed no signs of biliary obstruction. Abdominal CT revealed diffuse pancreatic swelling, edema of the duodenal bulb wall, and multifocal intestinal wall edema (Figure 2). Based on the diagnostic criteria–serum amylase exceeding three times the upper limit of normal and imaging findings–the patient was diagnosed with acute pancreatitis of moderately severe classification and unknown etiology. Following consultation with the gastroenterology department, management included fasting, discontinuation of therapeutic hypothermia, and continuous enemas to reduce intra-abdominal pressure. With this treatment, the patient’s intra-abdominal pressure decreased to 14 cmH2O by October 17. Gradual resumption of enteral nutrition began on October 20. A tracheostomy was performed on October 22. The patient was successfully weaned off ventilator support on November 11 and transferred to a rehabilitation hospital for further treatment on November 15.

### Follow-up and outcomes

The patient was followed up for 1 month after discharge. She continued rehabilitation therapy at the rehabilitation hospital. No further symptoms of pancreatitis occurred; however, the patient had not regained consciousness.

## Case 2

### Patient information

A 62-years-old male with a history of diabetes and coronary atherosclerotic heart disease was admitted at 20:00 on March 20, 2025, presenting with “left-sided limb weakness and slurred speech for 5 h.” A head CT performed at a local hospital 1 h after symptom onset showed no significant hemorrhage, leading to a diagnosis of cerebral infarction. The patient’s condition gradually worsened after intravenous thrombolysis with Alteplase (specific dose unknown). He was then transferred to our Advanced Stroke Center (Figure 1).

### Clinical findings

On admission to our center, physical examination revealed the patient was conscious with dysarthria. He had blindness in the right eye, a left pupil size of 0.25 cm with prompt light reflex, right gaze palsy, left-sided muscle strength of grade 2, right-sided muscle strength of grade 5, a positive left Babinski sign, and a negative right Babinski sign. The NIHSS score was 12.

After discussing the condition with the family, a neurologist performed cerebral angiography at 01:00 on March 21, 2025, which revealed occlusion distal to the C7 segment of the right internal carotid artery. Thrombectomy was subsequently performed. Post-procedure, the patient’s level of consciousness gradually deteriorated, and the left pupil dilated to 0.4 cm. An emergency CT scan showed significant right cerebral edema with midline shift exceeding 1 cm. Following neurosurgical consultation, a decompressive craniectomy was performed at 16:00 on March 21, 2025. Postoperatively, the patient was transferred to the ICU for advanced life support.

### Diagnostic assessment

The patient was diagnosed with cerebral infarction and cerebral edema.

### Therapeutic intervention

The patient was admitted in a comatose state with a Glasgow Coma Scale score of 5 and underwent therapeutic hypothermia, with body temperature regulated to a target range of 33 °C–34 °C based on bladder core temperature monitoring.

On March 22, a nasoenteric tube was placed for post-pyloric feeding.

On March 23, intravesical pressure was measured at 20 cmH2O. Management included reducing the enteral nutrition infusion rate and administering enema therapy.

**FIGURE 3:**
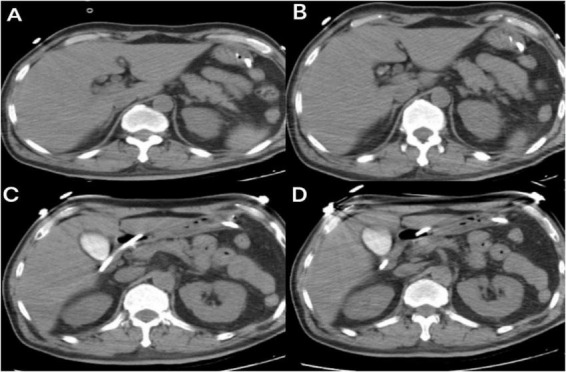
Non-contrast computed tomography of the pancreas in Case 2 reveals focal swelling of the pancreas (in the tail region), as shown in axial images **(A–D)**.

On March 24, intravesical pressure increased to 25 cmH2O. Laboratory tests showed elevated serum amylase at 400 U/L (reference range: 35–135 U/L). Bedside ultrasound indicated peri-pancreatic exudation, suggestive of possible pancreatitis. Abdominal computed tomography (Figure 3) and ultrasound revealed no signs of biliary obstruction, and serum triglycerides were 0.75 mmol/L (reference range: <1.70 mmol/L), within the normal range. After initiating fasting and other supportive treatments, intravesical pressure remained persistently above 20 cmH2O on March 25. Considering a potential association with therapeutic hypothermia, rewarming to 35 °C was initiated. Subsequently, serum amylase decreased to 256 U/L (reference range: 35–135 U/L), lipase was 334 U/L (reference range: 1–60 U/L), and intravesical pressure dropped to 14 cmH2O. The patient’s serum lipase exceeded three times the upper limit of normal, and ultrasound indicated pancreatic exudation, leading to a diagnosis of acute pancreatitis (mild).

Due to persistent significant cerebral edema, targeted temperature management at 33 °C was resumed on March 27.

However, by March 28, serum amylase increased again to 442 U/L (reference range: 35–135 U/L), lipase was 243 U/L (reference range: 1–60 U/L), and intravesical pressure rose again to 22 cmH2O. The patient’s condition improved once more following rewarming.

Consequently, the therapeutic hypothermia protocol was discontinued. Despite the complex clinical course, the patient was successfully weaned off mechanical ventilation on April 5 and transferred to a rehabilitation hospital for further care.

### Follow-up and outcomes

Follow-up after discharge revealed no further symptoms of pancreatitis. The patient recovered well, was alert and oriented, and had begun functional rehabilitation exercises.

## Discussion

Therapeutic hypothermia has achieved significant efficacy in the management of severe traumatic brain injury. Concurrently, increasing attention is being paid to the growing incidence of complications as core body temperature decreases, particularly among elderly patients and those with multiple comorbidities. Our center observed two consecutive cases of acute pancreatitis occurring during therapeutic hypothermia, which we believe warrant reporting. According to the Atlanta classification (5), acute pancreatitis was definitively diagnosed in both patients. Although neither case progressed to severe pancreatitis, the complication substantially prolonged their hospital stay. We organized a multidisciplinary discussion involving gastroenterology, neurosurgery, and radiology to explore the etiology. Here, we present these two cases and discuss the potential causes and preventive measures.

The etiologies of acute pancreatitis are diverse; however, hypothermia-induced acute pancreatitis is rarely reported. A literature search yielded two intriguing articles. One reported that pancreatitis is frequently found during autopsies of hypothermia victims (6). Another article stated that hypothermia-induced pancreatitis is common, with autopsy findings present in 20%–30% of cases, and that mildly elevated serum amylase without clinical evidence of pancreatitis is even more common, occurring in 50% of cases in one study (7). Therefore, we suspect that the acute pancreatitis in our two cases may be associated with therapeutic hypothermia. Current evidence for hypothermia-induced pancreatitis primarily comes from autopsy findings, with no established gold standard for diagnosis. Thus, we can only posit its possibility. The pancreatitis in these two cases was not severe and improved upon rewarming. However, other potential causes, such as stress and medication, must be excluded.

Both patients were neurocritical care cases. Severe neurological injury often triggers a potent stress response that can damage multiple organ systems. The most common manifestation is stress ulceration in the digestive tract, which can lead to gastrointestinal mucosal injury and decreased motility. The gut is not only the “central organ” first affected by stress but also a potential “initiating organ” for multi-organ dysfunction (8). Injury to the intestinal mucosa can result in upper gastrointestinal bleeding, a common clinical observation. In the lower digestive tract, it can lead to bacterial translocation and reduced motility, affecting the entire digestive system, including the pancreas (9). Therapeutic hypothermia may exacerbate gastrointestinal injury, as intestinal peristalsis weakens during treatment, potentially leading to paralytic ileus (7). Therefore, in these two cases–especially given their early onset–a stress component cannot be ruled out. The use of dexmedetomidine for stress modulation during hypothermia is crucial (10).

We also considered whether enteral nutrition was initiated too early. Current Chinese guidelines for neurocritical care recommend initiating enteral nutrition within 24–48 h (11), which was followed in both cases. For patients undergoing hypothermia, placing a nasoenteric tube is advised to reduce aspiration risk (12). Under these conditions, drug absorption via the oral or nasoenteric route may be impaired, and thus this route should be avoided. Furthermore, the entire gastrointestinal tract may exhibit petechial hemorrhages. Liver function may be compromised, possibly due to reduced cardiac output, while decreased lactate clearance can contribute to acidosis (7). Current evidence on the timing of enteral nutrition is controversial, though most guidelines favor early initiation. However, given the impaired intestinal function during hypothermia, the timing should be individualized, with close monitoring of abdominal signs post-initiation.

Our literature review revealed another potential cause of acute pancreatitis: drug-induced pancreatitis. Drug-induced acute pancreatitis is generally considered rare (13). Most evidence comes from case reports relying on exclusion diagnosis, supported by details such as drug dosage, timing relative to symptom onset, response to withdrawal, and recurrence upon rechallenge (14, 15). Among drugs potentially implicated (14, 15), we identified one common to both patients: valproic acid (16). Proposed mechanisms for valproic acid-induced pancreatitis include: ➀ direct toxicity mediated by oxygen free radicals, possibly due to depletion of free radical scavengers like peroxidase and superoxide dismutase; ➁ accumulation of toxic metabolites or intermediates; and ➂ immune responses (15). Furthermore, Badalov et al. (17) (New York, 2007), in their evidence-based classification and review of drug-induced acute pancreatitis, categorized valproic acid as a Class Ia agent (strong association with documented rechallenge). In patients with severe brain injury, we administer valproic acid via continuous intravenous infusion (0.8 g/day) for seizure prophylaxis. Our center has no prior documented cases of valproic acid-induced pancreatitis. In these two cases, pancreatitis gradually improved without discontinuing valproic acid, leading us to conclude it was not the primary suspected etiology. Nevertheless, in such clinical scenarios, drug-induced factors should be considered and vigilantly monitored.

### Take-away

Particular attention should be paid to abdominal signs during therapeutic hypothermia, and acute pancreatitis should be suspected in patients presenting with abdominal distension. If symptoms of pancreatitis arise, early rewarming and discontinuation of hypothermia therapy are warranted. Furthermore, anti-stress therapy during hypothermia is crucial.

This study has several limitations. First, as a case report involving only two patients, the findings cannot be generalized to a broader population, nor can a definitive causal relationship between therapeutic hypothermia and acute pancreatitis be established. Second, due to the retrospective observational nature of the study, we were unable to fully account for all potential confounding factors, such as individual variations in stress response, potential effects of concomitant medications (e.g., valproate sodium), and intestinal functional status. Additionally, diagnosing acute pancreatitis in comatose or sedated patients is hindered by the absence of subjective complaints such as abdominal pain. Future research should include larger-scale prospective studies or case-control studies to clarify the incidence, risk factors, and pathophysiological mechanisms of hypothermia-associated pancreatitis.

## Conclusion

Acute pancreatitis during therapeutic hypothermia is exceedingly rare and is likely attributable to multifactorial causes, including hypothermia itself and the stress response. Nevertheless, hypothermia is believed to play a significant role in its pathogenesis. Therefore, vigilance for this complication is essential during therapeutic hypothermia to avoid delays in diagnosis and management. Close monitoring of abdominal signs is mandatory. We recommend routine monitoring of intra-abdominal pressure. Should abdominal distension occur, serum amylase and lipase levels should be checked, and a protocol for gradual rewarming should be considered.

## Data Availability

The raw data supporting the conclusions of this article will be made available by the authors, without undue reservation.
